# Anatomic, histopathologic, and echocardiographic features in a dog with an atypical pulmonary valve stenosis with a fibrous band of tissue and a patent ductus arteriosus

**DOI:** 10.1186/s13028-017-0314-z

**Published:** 2017-07-11

**Authors:** Hakyoung Yoon, Jaehwan Kim, Sang-Soep Nahm, Kidong Eom

**Affiliations:** 1 Department of Veterinary Medical Imaging, College of Veterinary Medicine, Konkuk University, 120, Neungdong-ro, Gwangjin-gu, Seoul, 143-701 Republic of Korea; 2 Veterinary Medical Teaching Hospital, Konkuk University, Seoul, 143-701 Republic of Korea; 3 Laboratory of Veterinary Anatomy, College of Veterinary Medicine, Konkuk University, 120, Neungdong-ro, Gwangjin-gu, Seoul, 143-701 Republic of Korea

**Keywords:** Anatomy, Echocardiography, Fibrous band of tissue, Histopathology, Patent ductus arteriosus, Pulmonary stenosis

## Abstract

**Background:**

Congenital pulmonary valve stenosis and patent ductus arteriosus are common congenital heart defects in dogs. However, concurrence of atypical pulmonary valve stenosis and patent ductus arteriosus is uncommon. This report describes the anatomic, histopathologic, and echocardiographic features in a dog with concomitant pulmonary valve stenosis and patent ductus arteriosus with atypical pulmonary valve dysplasia that included a fibrous band of tissue.

**Case presentation:**

A 1.5-year-old intact female Chihuahua dog weighing 3.3 kg presented with a continuous grade VI cardiac murmur, poor exercise tolerance, and an intermittent cough. Echocardiography indicated pulmonary valve stenosis, a thickened dysplastic valve without annular hypoplasia, and a type IIA patent ductus arteriosus. The pulmonary valve was thick line-shaped in systole and dome-shaped towards the right ventricular outflow tract in diastole. The dog suffered a fatal cardiac arrest during an attempted balloon pulmonary valvuloplasty. Necropsy revealed pulmonary valve dysplasia, commissural fusion, and incomplete opening and closing of the pulmonary valve because of a fibrous band of tissue causing adhesion between the right ventricular outflow tract and the dysplastic intermediate cusp of the valve.

**Conclusions:**

A fibrous band of tissue between the right ventricular outflow track and the pulmonary valve should be considered as a cause of pulmonary valve stenosis. Pulmonary valve stenosis and patent ductus arteriosus can have conflicting effects on diastolic and systolic dysfunction, respectively. Therefore, beta-blockers should always be used carefully, particularly in patients with a heart defect where there is concern about left ventricular systolic function.

## Background

Congenital pulmonary valve stenosis (PS) and patent ductus arteriosus (PDA) are common congenital heart defects in dogs [1]. PS is a potentially fatal disease and has three principal components: a dome-shaped valve with commissural fusion, a dysplastic (thickened) valve with or without hypoplasia of the leaflets, and a hypoplastic pulmonary annulus (with an aortic annulus to pulmonary annulus diameter ratio of >1.2) [2, 3]. PDAs are classified into four types, i.e., type I (gradually tapered), type IIA and IIB (abruptly narrowed), and type III (non-attenuated) according to the diameter of the PDA [4]. If severe enough, a PDA can cause congestive heart failure.

To date, only nine canine cases of concurrent PS and PDA have been reported [1, 5], and there is no detailed description of the anatomic, histopathologic, and echocardiographic findings in atypical PS with a concomitant fibrous band of tissue and PDA in a dog. Multiple heart defects with complicated haemodynamics make these anomalies difficult to assess [6].

This report describes the anatomic, histopathologic, and echocardiographic features in a dog that had concomitant PS with atypical valve dysplasia including a fibrous band of tissue and PDA.

## Case presentation

A 1.5-year-old intact female Chihuahua dog weighing 3.3 kg was presented with a cardiac murmur and intermittent cough. The owner reported that the dog also had poor exercise intolerance. Auscultation revealed a grade V/VI crescendo-decrescendo right-sided murmur and a continuous grade VI left basilar machinery murmur. Systolic blood pressure was 150 mmHg. Thoracic radiography revealed an increased vertebral heart score (11.3; reference range 8.7–10.7 [7]), sternal contact (4.5; reference range 2.5–3.0 [8]), and a bulge at the cranial aspect of the cardiac silhouette on the lateral view. Generalized cardiomegaly, a reverse D-shaped heart, and a bulge at 1–2 o’clock of the cardiac silhouette were identified on the ventrodorsal view.

**Fig. 1:**
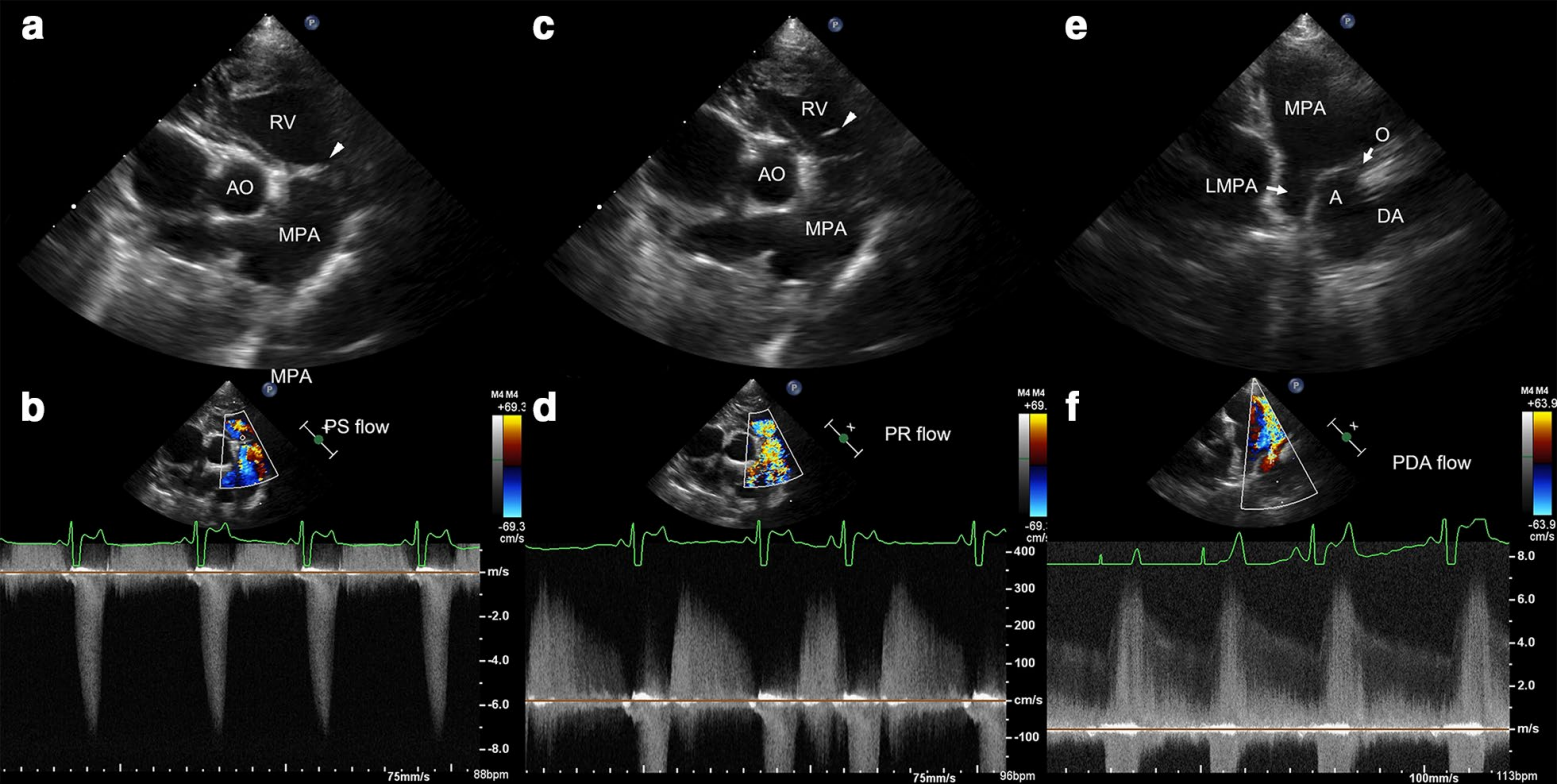
Echocardiographic images of valvular pulmonary stenosis and patent ductus arteriosus. **a** Right parasternal short-axis (RPSA) image acquired at the heart base level showing right ventricular concentric hypertrophy with dysplastic and a fused pulmonary valve during systole. **b** Continuous-wave Doppler image showing pulmonary stenosis (PS) with a peak transpulmonary velocity of 7.3 m/s and a pressure gradient (PG) of 213 mmHg. **c** RPSA image showing prolapse towards the right ventricle during diastole. **d** CWD image showing a peak diastolic pulmonary regurgitation (PR) velocity of 3.3 m/s and a PG of 44 mmHg. **e** Left parasternal long-axis image showing a patent ductus arteriosus (PDA). **f** At the same view, a CWD image showing left-to-right shunt flow with a peak systolic PG of 144 mmHg and an end-diastolic PG of 59 mmHg. *A* ampulla of patent ductus arteriosus, *AO* aorta, *DA* descending aorta, *LMPA* left main pulmonary artery, *MPA* main pulmonary artery, *O* orifice of patent ductus arteriosus facing main pulmonary artery, *RV* right ventricle

Transthoracic echocardiography revealed severely dysplastic pulmonary valve (PV) stenosis characterized by suspected commissural fusion with a late peak systolic velocity of approximately 7.3 m/s corresponding to a transpulmonary systolic pressure gradient (PG) of 213 mmHg (Fig. 1a, b). A normal PV annulus (11 mm compared with an aortic valve annulus of 12.5 mm) was identified. The PV showed diastolic doming toward the right ventricle with severe pulmonary regurgitation (PR) of 3.3 m/s and a PG of 44 mmHg (Fig. 1c, d). In addition, there was severe concentric hypertrophy of the right ventricle with a hyperechoic right ventricular endocardium and systolic and diastolic septal flattening. Ductal flow along the left wall of the main pulmonary artery (MPA) was identified due to left-to-right flow of the PDA with a systolic PG of 144 mmHg and a diastolic PG of 59 mmHg (Fig. 1e, f). Evidence of a reverse PDA was not found on a bubble study. The size of the PDA duct was approximately 2.2 mm. The pulmonary (Qp) and systemic (Qs) flow ratio was 1.5 (reference range 0.71–1.29 [9]).

An end-diastolic volume index of 145 ml/m^2^ and an end-systolic volume index of 42 ml/m^2^ were obtained by a modified Simpson’s rule formula. In addition, the following parameters were acquired: fractional shortening = 39.1%, LA: AO = 1.5; MPA: AO = 0.8; E = 82 cm/s; A = 51 cm/s; E: A = 1.6; IVRT = 50 ms; E: IVRT = 1.6; S′ = 8.4 cm/s; E′ = 5.5 cm/s; A′ = 6.6 cm/s; and tricuspid annular plane systolic excursion = 6.3 mm. No evidence of mitral, tricuspid, or aortic valve regurgitation was identified.

**Fig. 2:**
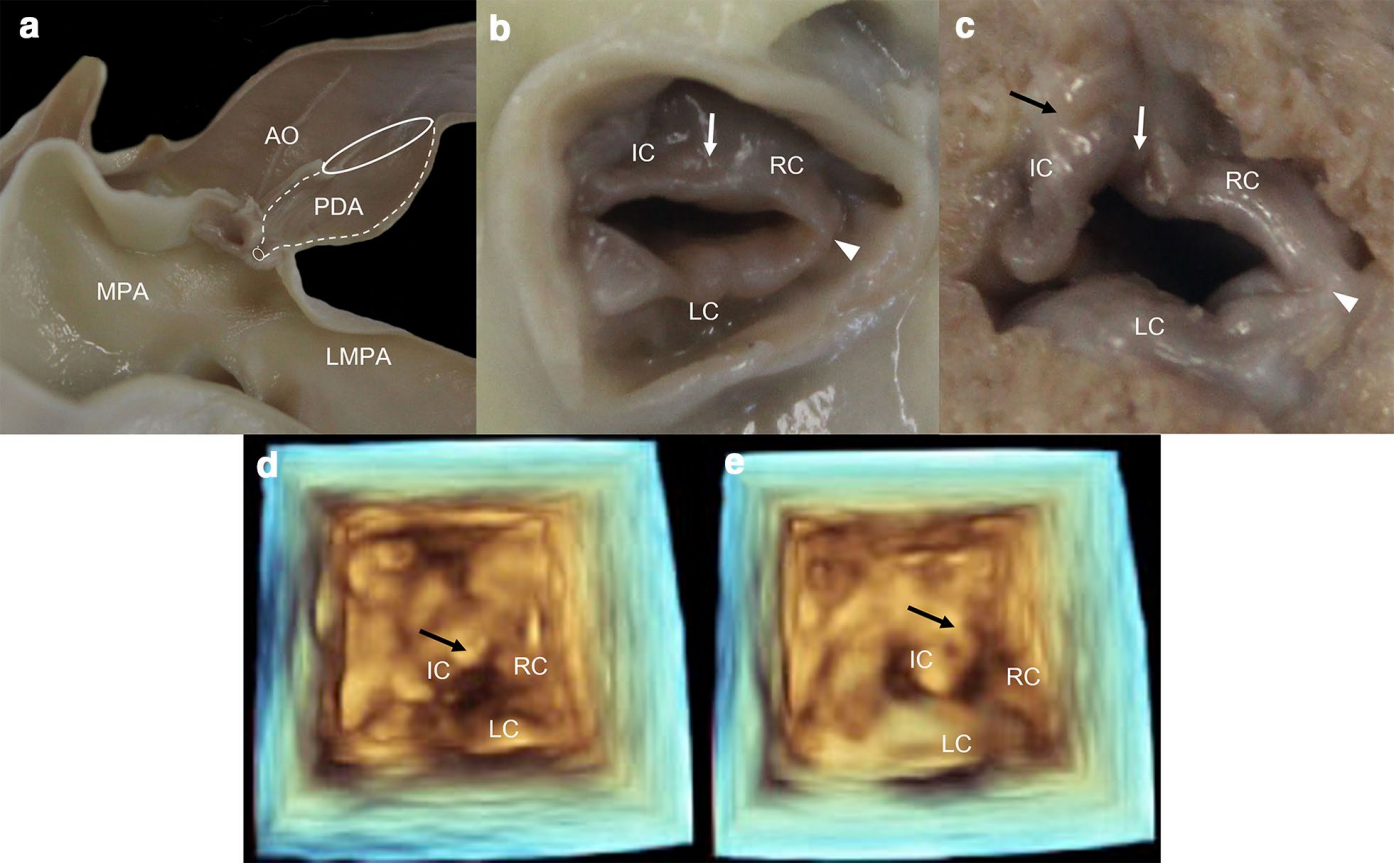
Post mortem anatomic and three-dimensional echocardiographic images. **a** Main pulmonary artery (MPA) bulge by post-stenotic dilation and patent ductus arteriosus (PDA). **b** Pulmonary valve viewed from the main pulmonary artery. The intermediate cusp (IC) and right cusp (RC) (*white arrow*) as well as the right cusp (RC) and left cusp (LC) (*white arrow head*) are completely fused and all pulmonary cusps are very thickened. **c** Pulmonary valve viewed from the right ventricle. Between the right ventricular outflow tract and the IC, adhesion by fibrous tissue (*black arrow*) has contributed to the IC pointing back towards the right ventricular outflow tract. Three-dimensional echocardiographic images in systole (**d)** and diastole (**e)** show limited pulmonary valve motion. Adhesion by fibrous tissue (*black arrow*) is observed as a structure protruding into the right ventricular outflow tract in both systole and diastole

On three-dimensional (3D) echocardiography, the PV was dysplastic (thickened) and misshapen, showing incomplete opening and closing in systole and diastole (Fig. 2d, e). Interestingly, a protruding structure connecting the intermediate cusp (IC) and right ventricular outflow tract (RVOT) was identified. However, it was impossible to differentiate the structures.

Diagnoses of right ventricular concentric hypertrophy, severe pulmonary stenosis with valve dysplasia without annular hypoplasia and with PR, and left-to-right shunting by a PDA were made on echocardiographic examination. At that point, a one-month course of a beta-blocker was started. The decision was also taken to insert a 4 mm Amplatz canine duct occluder (ACDO) and perform a balloon pulmonary valvuloplasty (BPV). The ACDO was successfully introduced through the left femoral artery prior to BPV. The BPV procedure was attempted through the ipsilateral femoral vein. A continuous IV lidocaine infusion of 50 µg/kg/min was maintained during the BPV. An IV bolus of lidocaine 2 mg/kg was injected when attempting to advance the guide wire and catheter sheath from the right ventricle to the right atrium. Insertion of the guide wire and catheter sheath into the MPA was unsuccessful because of the thick ventricular wall and narrow cavity. During several attempts at insertion of the guide wire and catheter sheath, acute cardiac arrest without ventricular fibrillation was detected on electrocardiography and auscultation. External cardiac compression (10 times per 5 s) and manual ventilation (every 5 s) were performed and atropine 0.04 mg/kg and epinephrine 0.02 mg/kg were injected intravenously every 5 min. Transcutaneous electrical pacing was unavailable so was not performed. Despite our efforts, the dog succumbed to cardiac arrest, but was donated for research purposes. Upon removal of the heart and great vessels at necropsy, bulges were identified in the MPA and PDA. After removal of the ACDO device, the aorta, PDA, MPA, and left main pulmonary artery were hemisected longitudinally.

The PDA was determined to be type IIA, characterized by a marked distal narrowing in the diameter of the duct of more than 50% (Fig. 2a) [4]. The size of the PDA orifice facing the MPA was approximately 1.7 mm on necropsy and approximately 1.9 mm on angiography, and smaller than the diameter of 2.2 mm seen on echocardiography.

**Fig. 3:**
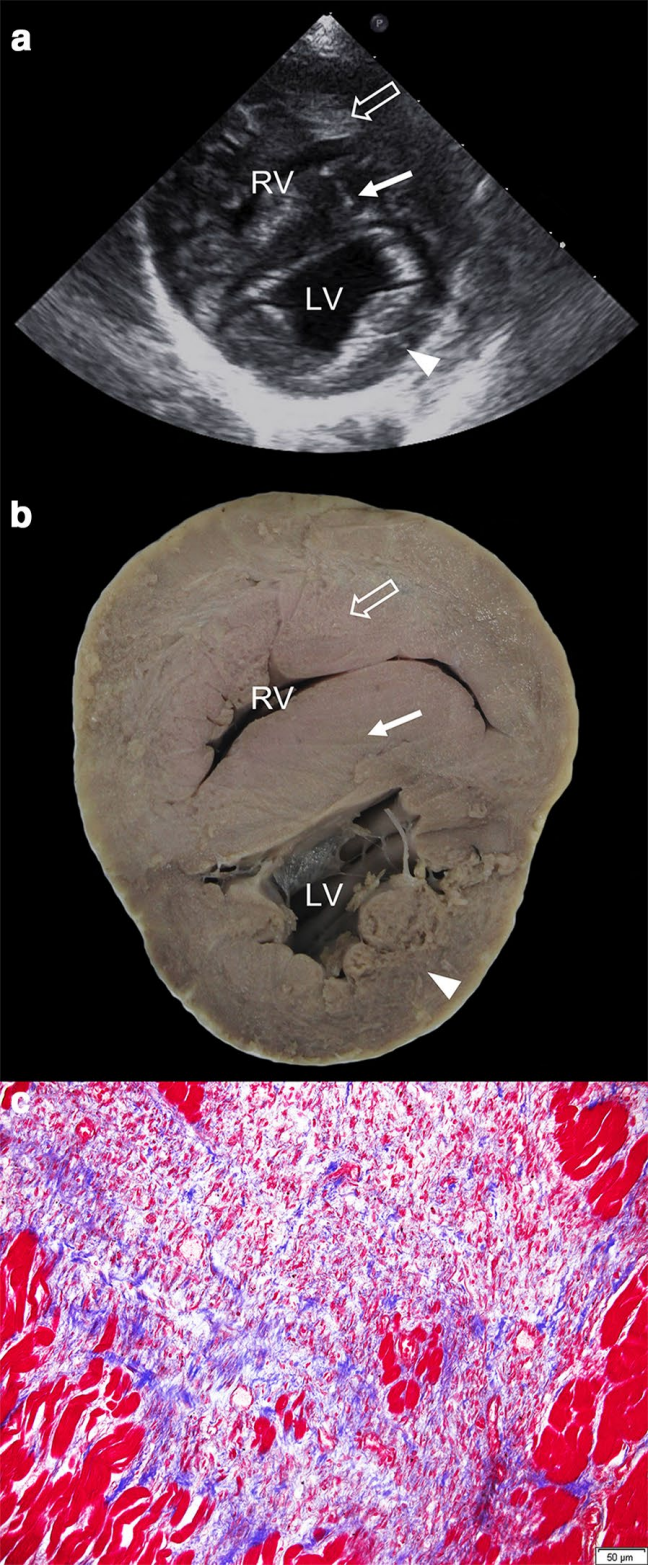
Echocardiographic, anatomic, and histopathologic images of the ventricular wall. The right ventricular (*blank arrow*) and septal (*white arrow*) wall is thicker than the left ventricular wall (*arrow head*) on a right parasternal echocardiographic short axis image (**a**) and a transverse gross image showing irreversible septal flattening (**b**). **c** Histopathologic findings for the right ventricle stained with Masson’s trichrome protocols. Microscopic views at 200× magnification show fibrosis that is stained blue because of the collagenous tissues. RV, right ventricle; LV, left ventricle

The PV was dysplastic and appeared bicuspid because the IC and right cusp were hypoplastic and fused (Fig. 2b). In addition, an adhesion caused by a suspected fibrous band of tissue was identified between the IC and RVOT (Fig. 2c), causing the former to point backward towards the latter. Transection at the papillary level of the left and right ventricles revealed severe concentric RV hypertrophy and irreversible septal flattening (Fig. 3b).

**Fig. 4:**
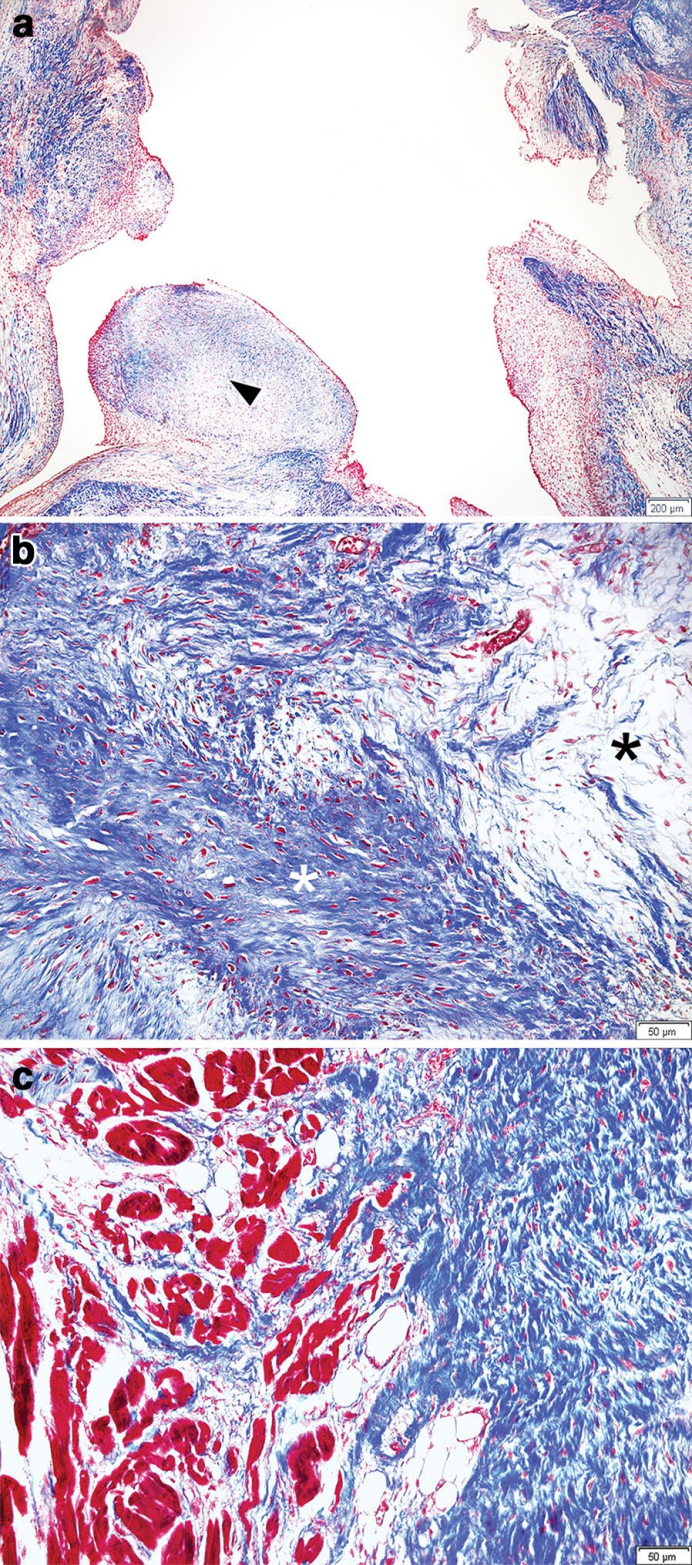
Histopathologic findings of the pulmonary valve stained with Masson’s trichrome protocols. **a** Microscopic view at 40× magnification shows varying degrees of fibrosis in the pulmonary valve including granulomatous tissue (*arrow head*). **b** Microscopic view of 200× magnification shows mixed patterns of dense fibrotic tissue (*white asterisk*) and loose fibrotic tissue (*black asterisk*). **c** Microscopic view of 200× magnification shows fibrotic tissue that make an adhesion between right ventricular outflow track and pulmonary valve

The PV valve, RV wall, ventricular septal wall, and left ventricular wall were then stained using haematoxylin-eosin and the Masson’s trichrome method. The tissue connecting the PV valve and the RV wall was identified as dense mature fibrous tissue consisting of collagenous fibres (Fig. 4c). The inner margin of the PV valve also consisted of dense mature fibrous tissue (Fig. 4a), but the inner area of the valve consisted of loose immature fibrous tissue (Fig. 4b). Fibrous tissues containing collagenous material were identified multifocally in the right ventricle and septal wall (Fig. 3c).

## Conclusions

In this case, the PS took the form of a dysplastic valve with a fibrous band of tissue but without annular hypoplasia. The fibrous band of tissue caused severe PS with a PG of 213 mmHg (severe PS > 80 mmHg [2]) and PR with prolapse of the PV. Although dome-shaped prolapse of the PV in diastole can be caused by ductal flow of a PDA [10], the PV being forced backwards by the fibrous band of tissue could have been a contributing factor.

On the basis of anatomic, histopathologic, and echocardiographic evaluation, the PR could have become worse for the following reasons: the PV pointing backward towards the RVOT because of the fibrous band of tissue; incomplete closing of the PV because of a dysplastic valve; and prolapse towards the right ventricle due to ductal flow in the PDA [10].

**Fig. 5:**
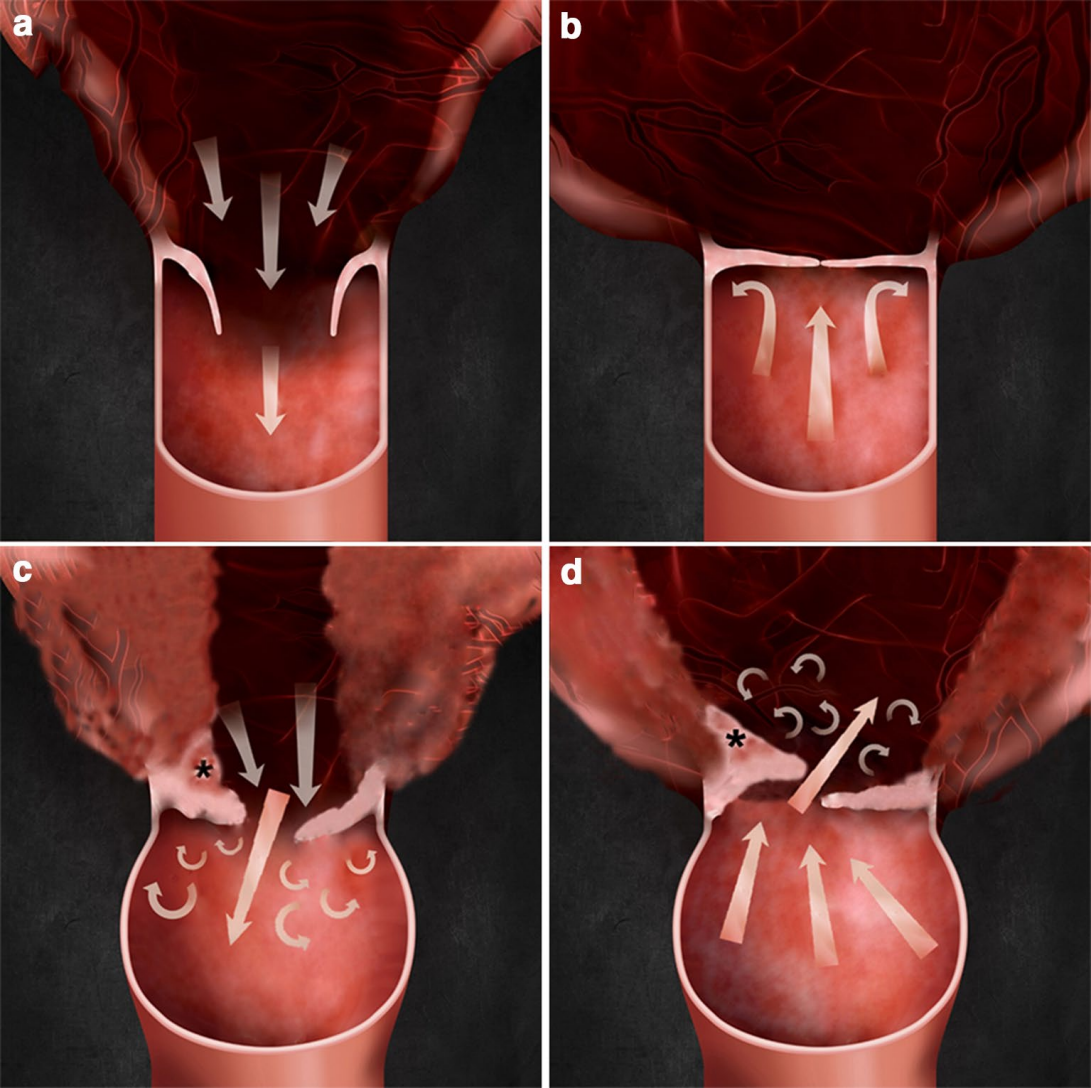
A normal pulmonary valve and an abnormal dysplastic valve with a fibrous band of tissue. **a** Opening of normal pulmonary valve in systole. **b** Closing of normal pulmonary valve in diastole. **c** Incomplete opening of dysplastic pulmonary valve with fibrous band of tissue in systole. **d** Incomplete closing of dysplastic pulmonary valve with fibrous band of tissue in diastole. *Asterisk* indicate a fibrous band of tissue

This case report suggests that a fibrous band of tissue as well as fusion and dysplasia of the PV [3] can affect the severity of PS. The fibrous band of tissue at the PV forcing the IC towards the RVOT in systole and diastole may have contributed to the limited motion of the valve (Fig. 5). Three-dimensional echocardiography was performed to investigate the misshapen valve but the images were structurally distorted such that the separate parts of the valve appeared to be fused. Further 3D echocardiographic studies of the PV in dogs are needed.

A differential diagnosis of pulmonary hypertension (PH) was required because of a PR PG of 44 mmHg. However, evidence of PH was considered to be insufficient because of the lack of dilation of the pulmonary artery in the caudal lung field on radiography and left-to-right PDA flow with a systolic PG of 144 mmHg and a diastolic PG of 59 mmHg on echocardiography. In addition, ductal flow toward the PV from the PDA duct could accelerate the velocity of the PR. Therefore, determination of PH by PR in dogs with PDA should be made by comprehensive evaluation of bidirectional, very low, or reverse PDA flow and a decreased pulmonary to systemic flow ratio [11].

To date, the threshold Qp/Qs causing significant hemodynamic changes in the presence of a PDA is unclear, but has been reported to be 1.5 in dogs with a ventricular septal defect [12]. The Qp/Qs was also 1.5 in the present case, and the hemodynamic significance of this is unclear. However, an elevated end-diastolic volume index, end-systolic volume index, and LA:AO ratio without mitral regurgitation could suggest a significant hemodynamic change caused by the PDA [11]. The ACDO method has been recommended for correction of a PDA rather than surgical ligation because BPV should be performed concurrently [13].

However, PS and PDA are difficult to manage at the same time because of the decreased systolic function caused by the PDA [11] and decreased diastolic function caused by the PS. Severe PS requires administration of a beta-blocker to decrease cardiac contractility, heart rate, and myocardial oxygen consumption [13]. This may cause a further decrease in systolic function to below the normal range in patients with PDA. Therefore, beta-blockers should always be used carefully, particularly in patients with a heart defect where there is concern about left ventricular systolic function. In the present patient, the echocardiographic findings for PS were more severe than those for PDA, hence the prescription of a beta-blocker for a month. There were no clinical signs of exercise intolerance, respiratory disorder, syncope, or depression after one-month course of a beta-blocker. The PDA procedure was performed prior to the PS procedure because the PDA can be repaired within a short time and the procedure is easier to perform than that for PS. A PS procedure can cause severe arrhythmia or cardiac arrest when the approach to the MPA is delayed. PDA shunt flow during this time can increase the risks of the procedure. Therefore, performing the PS procedure after correction of the PDA could produce a more stable result.

In this study, an approach via the femoral vein was used because the angle of entry for the guide wire was more gradual than it would have been through the jugular vein, and the femoral artery was required for the ACDO procedure and was adjacent to the femoral vein [13]. However, the dog succumbed to a cardiac arrest during insertion of the guide wire and balloon catheter. Repeated stimulation of the heart can cause a fatal arrhythmia or cardiac arrest, so extreme care is needed during a BPV procedure [13].

The loose immature fibrous tissue seen inside the PV tissue on histopathologic examination suggested progressive thickening of the PV, resulting in more severe PS. Therefore, delaying therapy after a diagnosis of PS could worsen the severity of the condition. Multifocal fibrous tissues were identified in the right ventricle and septal wall, suggesting chronic ischemic injury because of the muscle thickening. Diseases involving cardiac fibrosis, such as in this case, require further study, including strain analysis using speckle tracking echocardiography and magnetic resonance elastography [14].

This is the first report describing concurrence of PS and PDA in detail. Our canine patient showed atypical PV dysplasia with a fibrous band of tissue, hypoplasia, and fusion on anatomic, histopathologic, and echocardiographic evaluation. Concurrence of PS and PDA should be managed very carefully because these two entities have opposing effects on cardiac function. In addition, a fibrous band of tissue should be included as a cause of PV stenosis when a dome shape is seen moving toward the RVOT in diastole without PH in dogs. However, further studies are required to determine the aetiology of this phenomenon.

## Abbreviations

3D: three-dimensional
ACDO: Amplatz canine duct occlude
BPV: balloon pulmonary valvuloplasty
IC: intermediate cusp
MPA: main pulmonary artery
RVOT: right ventricular outflow tract
PDA: patent ductus arteriosus
PG: pressure gradient
PH: pulmonary hypertension
PR: pulmonary regurgitation
PS: pulmonary valve stenosis
PV: pulmonary valve
RA: right atrium
RV: right ventricular
